# Age is Nothing but a Number: Ben 10s, Sugar Mummies, and the South African Gender Order in the Daily Sun’s Facebook Page

**DOI:** 10.3389/fsoc.2021.706132

**Published:** 2021-09-29

**Authors:** Priscilla Boshoff, Ntombi Lina Mlangeni

**Affiliations:** Rhodes University, Makhanda, South Africa

**Keywords:** South Africa, sugar mummies, Ben 10, daily Sun, gender order, postfeminism

## Abstract

Stories about “Ben10” relationships between older women and their younger male lovers appear regularly in the *Daily Sun*, South Africa’s most popular tabloid newspaper. *Daily Sun* readers, who are typically township residents, engage vociferously over the rights and wrongs of such relationships on the tabloid’s Facebook page, and alternatively berate or support the older, working class women who feature in them. These women could be understood as “postfeminist” insofar as they are financially independent and sexually autonomous. Their actions echo those of the independent township women in the mid 20th century who, resisting patriarchal apartheid social engineering, brewed beer and rented rooms in order to assert their financial and sexual independence. In both cases, these women’s bold actions confront local hetero-patriarchal norms and call into question an ideal local patriarchal gender order. However, the meanings that are made by the readers of such women in Ben10 relationships today also reflect a social context characterised by a contestation over the meaning of rights, high rates of unemployment, gender-based violence and HIV, factors that curtail a premature diagnosis of postfeminist identity. Drawing on a textual analysis of several articles and their Facebook comments, we argue that any assessment of postfeminism in southern spaces must account for how historical and contextual factors such as these constrain the reach of global postfeminism.

## Introduction

This article explores the meanings of “Ben 10” relationships constructed in reports by the popular South African tabloid, the *Daily Sun*, and in the lively conversations that take place about these stories on the paper’s Facebook page. A township colloquialism, “Ben 10” refers to a man who enters into a sexual relationship with an older woman (a “sugar mummy”) on whom he is financially dependent. *Daily Sun* readers, who are typically township residents, engage vociferously over the rights and wrongs of such relationships as reported in the tabloid. An affront to local hetero-patriarchal norms, they call into question an ideal local patriarchal gender order which understands men as supporting their households as breadwinners and women as compliant and chaste sexual partners and mothers. Readers attempt to understand how these relationships challenge local gender norms, and alternatively defend or loudly decry the choices that are made by both the men and women involved.

However, their evaluations transcend simplistic frames of praise or blame. Some who support such relationships draw on the discourse of rights, as articulated in South Africa’s liberal constitution, to argue for sexual equality. But sexual equality, while undoubtedly an important facet of our post-transition milieu, cannot be taken for granted. Rights are themselves the subject of anxiety, and are not necessarily understood as providing an uncomplicated “freedom” for women to explore sex and sexuality, as endorsed by global postfeminist discourse - in this case, the “right” of older women to enjoy sex with younger men of their choice. This is because the right to freedom of sexuality is put into practice in a highly complex post-transition social milieu, characterised by the persistence of customary and Christian gender mores; high levels of gender-based violence; unemployment; and HIV prevalence. These factors emerge consistently within the Facebook discussions that comment on the *Daily Sun*’*s* Ben10 reportage, and inform our analysis. Yet these factors are not in themselves a sufficient explanation for the extreme opprobrium Ben10 relationships evoke. They complement a long-standing patriarchal anxiety attached to the “uncontrolled” sexuality of financially independent and older working-class women in township spaces. It is this history of backlash to non-conforming financial and sexual independence that provides the basis for the argument of this article.

## Postfeminism and the Older Woman

This issue of *Frontiers of Sociology* asks us to situate postfeminism and critical responses to the discourse within our African context. We start by asserting that critical approaches to postfeminism can expand our scope of understanding by examining older women in working class social settings in our southern context. This context complicates the premises on which the discourse of postfeminism depends and make visible the limits of its reach. Four contextual factors in South Africa appear particularly pertinent when considering older and working-class women in relation to postfeminism. First, despite Constitutional rights, many South African women still struggle to reach parity with men and realise the liberal second-wave feminist gains that postfeminist discourse takes as axiomatic. Then, far from women being financially independent, a necessary precondition for postfeminist consumption, very high rates of unemployment have profound implications for gender relations as a whole, as well as for women’s economic and social autonomy. Third, due to widely held customary and Christian mores, the overt display by women of sexuality and sexual desire which signals a postfeminist subjectivity is not always socially acceptable. Neither exposure to global media—including the digital sphere—where such identities are routinely celebrated, nor 2 decades of HIV politics and education which have forced sex and sexuality from the privacy of the bedroom into the public sphere, have been sufficient to dislodge this resistance entirely. Last but not least is the persistently high level of gender-based violence which curtails the personal freedom necessary for performing a postfeminist subjectivity.

These endemic issues are seemingly transcended by youthful and “modern” postfeminist performances online, such as women’s self-representations on Instagram (Bosch 2011; Iqani 2019). Their glamorous and sexualised consumption practices mark them as ideal global postfeminist subjects, much in line with Dosekun's (2015) “hyper-feminine” elite women in Lagos, Nigeria. But older working-class women in South Africa cannot be equated with older working-class women who appear in northern postfeminist scholarship. This goes beyond their being outside the class position which allows the uptake of the discourse through global flows of media. Their experiences and social location are too different. For this reason, we cannot draw on northern scholarship that examines popular culture depictions of older women’s sexuality in age-heterogenous relationships, such as the “cougar” women depicted in British tabloids (Hamid-Turksoy et al., 2014), celebrity tabloid news (Burema 2018) or Hollywood cinema (Whelehan 2013). Nor is the postfeminist critique of the makeover show (Tincknell 2011; McRobbie 2020) that scrutinises and reorders the older, working class woman’s body to maximise her sexual attractiveness and employability, of much use to us here. This scholarship examines how postfeminist discourse is mediated through popular culture forms, how it monitors the sexuality of the older woman, and inscribes her aging body as she constructs a disciplined postfeminist subjectivity in line with neoliberal demands within the declining welfare state.

Instead, we argue that we must begin by looking carefully at the specific social context in which postfeminist discourse attempts to take root. Especially pertinent to this study is a longstanding tension in the South African social fabric: the struggle between women who have historically attempted to assert themselves as independent economic and sexual subjects, and the hetero-patriarchies that have sought to control them. This history of gendered struggle must be understood within the context of colonial and apartheid racialised spatial segregation and the laws that controlled the movement of black South Africans into peri-urban black working-class residential areas, or “townships.”

We briefly set out this context below by drawing on anthropological and historical research into women’s independence and township sociality during the colonial and apartheid eras. It reveals the opprobrium attached to unmarried women who, living illegally in township spaces, won economic and social independence from customary patriarchal authority through beer brewing. Then, more recent scholarship on women in township settings foregrounds the repercussions of women claiming “rights” in a context of profound economic uncertainty. Beer brewing and rights and the gender conflicts these incite, shed light on the *Daily Sun*’*s* stories that deal with older, financially independent women and their relationships with Ben 10s in township settings.

## Beer-Brewing, Independent Women and Moral Panic

Colonial rule sought to harness black South African male labour for nascent local capitalism. To this end, men were allowed to live (primarily as migrant labour) in peri-urban “townships,” residential areas set aside for black residents adjacent to mines and urban industry. At the same time, black and white patriarchies colluded to exclude women from the formal economy (Delius and Glaser 2004; Freund 2007). While some women found work as semi-skilled labour in decentralised industries in the first half of the 20th century (Mager 1989; Minkley 1996) they more often found work as domestic labour (Cock 1980). Otherwise, women were expected to remain in rural areas under the authority of their fathers, or husbands and husbands’ families: only some married women could get permission to join husbands who lived permanently in town.

Despite the laws that sought to confine them to rural homesteads, young and other marginalised women—single “mothers,” “widows” and “deserted women” (Minkley 1996: 145)—moved to town when rural livelihoods became unsustainable, or the weight of patriarchal control unbearable (Minkley 1996; Freund 2007; Bank and Kamman 2011). These women survived in the informal economy by brewing beer, leasing living space and constructing households composed of women and children only (Minkley 1996; Hunter 2010; Bank 2011).

Beer brewing enabled unmarried women to assert an economic and sexual independence that was loudly decried by the (black and white) men who sought to control them (Freund 2007). Offering a vision of viable albeit ambiguous independence, beer-brewing profitably established “a class of independent women” who “succeeded their ambition to buy a wood and iron house” and became landladies “not under someone else’s rule” (Minkley 1996: 150). Unsurprisingly perhaps, beer-brewing was villainised and became a “discursive site for different fears and definitions about ‘the native family’ and the position and behaviour of black women in an urban environment” (Minkley 1996: 137–138). As a “women’s business” that made “real money” and enabled women to buy property, Minkley (1996: 151) describes how, in the city of East London, illicit home beer brewing by independent, unmarried women provoked a “moral panic” on the part of middle class black residents and white authorities, which appears to have centred on the women’s uncontrolled sexuality: a “myriad of wantonness” was “associated with the ‘presence of licentious and dangerously free females’ selling ‘illegal liquor.’” This included “‘illicit sex’, ‘fly-by-night lovers,’ ‘venereal disease’ and illegitimate children.” Indeed, the whole social fabric appeared to be at stake, including “rioting, fighting, crime, and juvenile delinquency” (Minkley 1996: 153; Bank and Kamman 2011).

The relative independence of this class of “stubborn, spirited and fiercely independent” women (Bank and Kamman 2011: 270) waned after the 1950s as apartheid legislation intensified the supervision of black South Africans in urban areas, and state and customary patriarchal control tightened its grip on women’s bid for independence: women were denied property rights, informal enterprises such as beer brewing were curtailed, and residence permits were given strictly to married women. Urbanised married couples were accommodated in formal state housing, constructed in keeping with the idea of a patriarchal, Christian and “modern” nuclear black family headed by a wage-earning husband (Hunter 2010). Women who wanted to stay in urban areas were thus entirely dependent on men: divorce, or the death of the husband, or father, could mean eviction and a return to rural homes.

## Unemployment, Rights and HIV: The Shifting Ground of Gender Relations

New constitutional rights in 1996 formally abolished men’s control over women, who reclaimed their “urban citizenship as independent agents in the city” (Bank and Kamman 2011: 271). Legislative changes had granted women certain property rights and a large number of single women gained access to low cost RDP housing provided by the South African state (Hunter 2010: 142). Women who have access to an RDP house and an independent income (such as through beer brewing) have more power to navigate intimate relations with men. However, these positive changes have taken place within a context of significant social upheaval, including increasing unemployment, violence and the HIV epidemic.

Hunter’s (2010) ethnographic work in a declining industrial township in KwaZulu-Natal explores the relationship between changing gender norms, unemployment, violence and HIV. He looks carefully at how the loss of male wages within the neoliberal economy reshaped customary ideals of masculinity; simultaneously, high unemployment and the discourse of rights encouraged women to forge new feminine identities. In neither case did these new masculinities and femininities escape the expectations of older, customary norms, as well as Christian ideals, with respect to gender relations. These reconfigured but still highly patriarchal gender relations are complexly connected to both gender-based violence and the high incidence of HIV (Hunter 2010; Jewkes et al., 2011; Morrell et al., 2012).

The effects of economic stagnation and chronic unemployment on gender relations cannot be overstated. Beginning in the mid-1970s, by 2019 unemployment measured 29,1%, an escalating crisis precipitated by South Africa’s entry into the global neoliberal economy. Mass unemployment ended the “patriarchal bargain” enabled by waged male labour, and undermined the dominant narrative of masculine success through work. This tension between the ideal of the working male breadwinner and the reality of South Africa’s poverty and unemployment within a wider context of rights has been referred to as a “crisis of masculinity” (Walker 2005). If waged labour had enabled customary marriage in which men provided for their wives and children, loss of income has meant that men find it difficult to fulfil the onerous financial obligations of *lobola* and the establishment and maintenance of a (polygamous) household. Instead, men with access to money support a number of sexual partners outside of marriage. Rather than “umnumzana” (in isiZulu, the head of a household), such men are described as “isoka,” a man with many sexual partners, defended as men’s custom. Notably, men without financial means are largely excluded from the sexual economy, as women, of whom far more are unemployed than men [43.5% of black women are unemployed in contrast to 35% of black men (Statistics South Africa, 2020)], expect material support within a sexual relationship.

Simultaneously, women have deployed the new discourse of rights in strategic ways to promote their economic and social interests (Hunter 2010). This includes the “right” to sexual pleasure and the freedom to have multiple sexual partners, as do men. Women also use rights discourse to argue for their “right to consume” desirable consumer goods (Posel 2004), as well as to live alone with their children [in other words, to be mothers who live independently, without men’s supervision, rather than be subordinated wives (Walker 1995)]. All these elements contribute to what in other spaces could be seen as a “postfeminist” identity, in which women define themselves as independent and sexually liberated, and signal a sexually attractive femininity through consumption (Gill 2007). However, reflecting on the ways in which the idea of the global postfeminist “It Girl” is taken up in township spaces, Hunter (2010: 131) reminds us that “(l)iberal rights can never be completely estranged from the wider, historically… constituted moral codes of ‘rights and wrongs.’” Consequently, women with more than one sexual partner, or who live independently, risk social censure and being labelled “isifebe” (a “loose” woman, a prostitute; a woman with excessive sex-drive). Moreover, these choices provide a pretext for violence, as men may attempt to “straighten” non-conforming and “disrespectful” women by “disciplining” them (Hunter 2010: 173).

## The Gender Order and Sexuality

Women, in other words, must construct their postfeminist identities in relation to the local patriarchal gender order and its normative expectations (Boshoff 2021a). Drawing on Raewyn Connell’s (2009) constructivist approach to gender, we argue that readers’ evaluations of women’s (postfeminist) sexual and material practices are rooted in and flow from local cultural ideals of masculinity and femininity. Importantly for our argument, Connell’s model specifically considers the violent social re-ordering initiated by colonialism in southern spaces as it “smashed” (Connell 2009: 92) and reconstituted local gender orders for its own ends, as noted in the context section above. The model proposes a hierarchy of variously subordinated and complicit masculinities, and “emphasised” and non-conforming femininities, which are positioned in relation to hegemonic masculinity whose social ascendency is established within a balance of forces (Connell 1987: 183). Significantly, Connell (1987) insists on the historical and social specificity of any assessment of gender relations, for what “counts” as hegemonic masculinity or emphasized femininity depends on context and the forces at play. A key element of hegemonic masculinity in township spaces is the ability to provide in material ways for a partner and children; but it also includes a virile (hetero) sexuality, “toughness” and the capacity for violence (Ratele 2010). In contrast, “emphasised” femininity is characterised, *inter alia*, as sexually receptive but chaste, “respectful” and submissive to men’s authority (Hunter 2010).

This has not always been the case, particularly in relation to women’s sexuality; historical and ethnographic evidence suggests that before Christian morality became entrenched, women’s sexuality was less controlled than today (Epprecht 2008). Colonial discourses, including Christianity, constructed African women’s sexuality as hyper-sexual, primitive, dirty and morally corrupting–and thus in need of control (McFadden 2003; Tamale 2011). Women’s sexuality and sexual expression consequently became “a key site through which women’s subordination is maintained and enforced in postcolonial Africa” (Tamale 2005: 9). Yet, very little work has been done on African women’s agency, in particular their sexual agency, and female sexuality, sexual desire and gratification are rarely the object of analysis (Arnfred 2004; Tamale 2011).

## Sample and Method

An analysis is offered of three Ben 10 stories published in *Daily Sun* between 2015 and 2017. They are typical of the reportage on this topic, and are purposely selected for the wide range of Facebook comments they provoke. The number of comments on the three articles altogether is 3,338. The analysis is arranged under the themes of democracy, rights and sex; violence and unemployment, and HIV. HIV is not dealt with in any one article, but emerges obliquely in comments within a range of reports. Using multi-modal critical and visual discourse analysis methods, we examine how the paper constructs the Ben 10 relationships in these instances, paying particular attention to the composition of the images and lexicalisation. This serves to contextualise the analysis of the readers’ Facebook comments, which follow the articles.

### Democracy, Rights and Sex

“I LIVE WITH MY BEN 10 AND MY HUBBY!” Woman takes a second husband for sex! opens in typical *Daily Sun* style, by humorously setting up the narrative premise: “THE HUBBY has the money. The Ben 10 has the hot 4–5 (penis).” It is immediately followed by its antithesis: “AND PUSELETSO HAS TOO MUCH LOVE FOR JUST ONE MAN.” This distinctive presentational mode of dramatic headlines and overt narrativisation should not be read as mere conformity to tabloid conventions; rather, it is a key strategy by means of which *Daily Sun* constructs and communicates the moral dimensions of the imagined community it forms together with its readership (Wasserman, 2010; Boshoff, 2017). In other words, it is designed to invite identification and debate. The introduction establishes the premise of the story, which concerns the conundrum Puseletso faces in maintaining a relationship with her two partners, her “customary” husband, and her “Ben10.” A photograph shows them standing on either side of Puseletso, looking affectionately at her as she smiles at the reader. The direction of their gaze, and her frank engagement with the reader establishes her as the report’s protagonist. The story sets out the grounds for her unusual decision: she explains that her husband of 15 years couldn’t “satisfy” her in bed, on account of a “chronic disease.” Pains are taken at the start of the report to construct her resolution to bring her younger lover into “her RDP” home as one that was carefully made, for it “wasn’t an easy decision.” She declares that she “hated the idea of cheating” on her husband, whom she “(loves) as much as (she) ever did”; moreover, she “felt guilty” about her “boyfriend.” For these reasons, she “decided to tell Petrus (her husband through customary marriage) about Kagiso.”

She contrasts her bravery with the behaviour of other women: as “a shebeen queen” (a beer-brewer) (the kind of person who, the word implies, would be intimately aware of township social mores), she describes how she “doesn’t like what she sees happening around her.” “Most women I know in my kasi (township) cheat on their husbands,” she explains. Moreover, “they do it in secret.” She compares this reprehensible behaviour with her own honesty: “I decided to live with both of my men faithfully.” However, her honesty has not earned her any favours with fellow township residents, who “told me it was a bad thing” and “called me names.”

The report also establishes the relationship between the two men, who, the report reassures the reader, “have separate rooms.” While Petrus the husband is “not completely happy with the situation” (he confides that he has “become the laughing stock of our kasi”), he has “accepted it,” “for the sake of his children.” For his part, Kagiso declares that Puseletso’s married status does not affect him: he “isn’t worried that his girlfriend is married,” and he is “a happy man.” A further comparison is set up between Kagiso and the husband, for Kagiso and Puseletso are trying to “raise money for our wedding,” a direct contrast to the customary marriage that pertains between Puseletso and Petrus.

Noteworthy is that Petrus has a “disability grant” and works as a gardener; but Puseletso, a “Shebeen queen,” is presumably financially independent of her husband and able to support Kagiso, who is described as “unemployed.” Indeed, Kagiso’s dependent position is signified in the photograph by the frilly, effeminate hat which is comically perched on his head; while he hugs Puseletso close to his body, the space that this creates between Petrus and Puseletso suggests the separation that now exists within their relationship, as one reader observes: “She loves the Ben 10 more, just look at the space between her and the husband.”

#### Facebook Comments Discussion

*Daily Sun*’*s* Facebook page can be understood as a public forum in which readers share opinions on the stories of the day. It complements the paper’s daily letters page by means of which readers participate in constructing the moral and social dimensions of the imagined community of “SunLand,” including its gender order (Smith and Adendorff 2014; Boshoff, 2021b). It is in this light that the online commentary must be assessed. Many comments posted in response to the story mockingly deride the appearance of the characters involved, especially the purported age of the younger lover. “Who is Ben 10 between the 2 because they all look like pensioners 2 me,” captures the sentiment of such posts. However, the joking has its limits, as one reader irritably asserts, “this ain no joke its a seious matr (serious matter) so stop laughn mxm (expression of disapproval).” Woven between the dismissive laughter is a serious thread of concern about gender relations—“There is need to rethink the structure of relationships”—which acknowledges an underlying issue that demands attention. There are two strands to this thread: one is concerned with what happens when contemporary patriarchal gender relations, which endorse multiple partners for men, are overturned by women claiming the right to act as men do; the second is interested in sex as an ingredient within healthy relationships.

In the first strand, commentators compare Puseletso’s contemporary arrangement with the local practice of polygamy, which is aligned with “custom.” Some condemn, while others point out what appear to be the positive aspects of Puseletso’s course of action. Those who applaud her decision to take a Ben 10 lover remark gleefully on the social norm that excuses multiple sexual partners for men, and assert that it’s high time women did the same: “Yes mama, it’s your time now. Men have been doing this since the beginning of time,” says one. “Good for her!” exclaims another, “These men must feel how some of the women feel about polygamy.” Angrily, one person calls out patriarchal double standards, observing that men “take 2 or even 4 wives and more but as soon as a woman does it she’s called a hoe (whore) Mxm,” and concluding that men are a “bunch of hypocrites.”

This argument turns on the idea of “democracy” and its implicit rights, which are not always seen favourably: as one commentator sarcastically remarks, “yah neh its Democracy.” Some quip caustically on the highly visible example set by the then South African President, Jacob Zuma, a polygamous husband: “i can see tha you are competing with Zuma!” and, “Good polygammy Zuma did t first now we are also doing t he is leading by example.” The latter commentator also remarks that polygamy “is legal in SA,” a point taken up by another who exclaims that all unions need to be treated equally: “Polygamy is favoured by our parliament; therefore polyandry must be favoured!” This commentator even offers to assist Puseletso: “I will be your Advocate Puseletso in registering both marriages legally,” declaring that “this is the 21st century. Equality must prevail!!!”

However, this positive reception is countered by a more pessimistic reading. The idea of such “equality” is shocking to many, visible in comments that remark on it by means of the idiomatic expression “50 50.” This phrase appears in Hunter’s (2010: 132) ethnography, where he observes how women argue that “in a 50/50 world, they now have the right to have multiple partners, just like men.” Thus, one commentator exclaims in amazement, “Yhoooo nahh (expression of disbelief) I’ve never seen this in my life, yhooo ke 50 50 str8 (it’s 50 50 straight)”; while another remarks that “becoz its 50 50 thus y this lady manage to get two mens lyk (just as) men take two wifes huh.” “This is taboo in our culture” continues the first. Male readers in particular feel that it is impossible for two men to live under one roof: “yoh how can I sleep knowing my wife is having sex with another man?” asks one, while another remarks “Poo pedi sakeng tlhe banna (two bulls in one sack; i.e., two men under one roof), this is too much.” The use of the vernacular idiom, which constructs men as rivals who cannot share a space (unlike women who are expected to do so in polygamous unions) drives home this reader’s point about custom - for him, Puseletso’s arrangement is simply immoral: “We are seriously losing our morals and values.” Another similarly condemns the arrangement, sternly adhorting the husband to end the relationship: lomama isifebe (this woman is promiscuous), lobaba wokuqala akayeke lomama (the husband should leave her).

The use of the word “isifebe” is contentious. It can mean a promiscuous woman or a prostitute (Hunter 2010: 180). However, in some township communities the word can also refer to a woman with an “excessive” sex drive. Notably, the “isifebe” label is used colloquially as a policing tool to ensure that women do not express desire or engage actively in sexual encounters with men, but are rather passive sexual participants. Sex and sexual pleasure is still seen as a male domain and sexual appetite as a masculine trait; for this reason, women who dare to explore their sexuality are termed “isifebe” or “loose.” The expression of sexual desire can thus be interpreted as the woman lacking “respect,” as suggested in the comment “That old woman should be ashamed of herself, she clearly has no respect.”

The discourse of “hlonipa” (respect) is a means of guiding customary social relations (Hunter 2010). However, the language of rights and new forms of sex talk have disturbed gendered and generational lines of authority denoted by the concept of “hlonipa” (Hunter 2010: 138; Posel 2004). By taking another husband, Puseletso flouts custom, and thereby disrespects her first (legitimate) husband, under whose authority she is expected to reside. Respect is linked to adherence to customary values, but also to what counts as moral behaviour.

Discourses of immorality also frame women’s agency as alarming. Readers draw on Christian precepts to condemn the characters and their actions: “Ooooo!!! Lord wat have done to deserve this?” asks one amazed reader, while another exhorts “God” to “bring bck da ancient floods dat took place during da days of Aaron” for “we modern ppl (people) we r extremely sinning.” Indeed, for several readers, the story—an “ABOMINATION”—signals the “End of the world!!!!” Significantly, however, two comments grounded in Christian scripture present arguments defending Puseletso, and endorsing, rather than censuring, her subversion of patriarchal norms. Her actions are compared to those of Abraham’s “affair with his slave,” which is implicitly excused for “Sarah the wife was aware of it.” In this writer’s opinion, “They are a happy family,” and given their happiness, “who are we to judge them.” Another commentator similarly attempts to divert censure by quoting from the new testament: “let anyone without sin cast the first stone.”

A more pragmatic (but less visible) strand of argument puts aside morality in favour of a rational and “solutions” approach to the problem that is perceived in the couple’s sex life. The husband’s impotence is understood as an undoubted factor in Puseletso’s decision: “No sex, no love,” remarks a reader, while another agrees, “marriage without a good sex… doesn’t succeed.” Another goes as far as to indirectly blame the husband, for “this shows men that living an unhealthy life, leads our wifes to a unsatisfactory stage in our marriages.” “Let this be a lesson,” he warns. Lessons are indeed given, interestingly, in terms of biology, in which the “truth” about sexuality is pointed out: “nature is nature” remarks one. The phrase suggests that Puseletso has sexual needs which need to be met, an interesting inversion of the more common idea that it is men’s sexual drive that needs to be satisfied at all costs (Jewkes et al., 2005; Hunter 2010).

Another explains to other readers that “It’s indeed true that women reach their sexual peak in their 40’s,” and compares women’s sexuality to that of men who “lose their libido and sexual peak in that age, so it’s a disaster.” However, not everyone agrees with this gloomy conclusion; interesting here is a comment which points out that this is “normal human behaviour,” which should be accepted as such: “I am glad that there are women who are exploring their sexual nature without worrying about the pressure society puts on such normal human behaviour.” For this reader, and many others, Puseletso’s bold action renders her heroic: “Good woman, see there should be more women like her,” and “I want to be her,” express readers’ admiration. Her story also encourages other women who are not as bold or lucky: “I wish it could be me, she’s my role model nami ngifuna ukuba nesthembu (I want to be polyandrous too).” What is especially attractive to these readers is Puseletso’s authority: “I like her courage” declares one, while another admiringly asserts that “This lady is a straight up boss. I want to be like her.”

### Violence and Unemployment

EVIL BEN 10 KILLED OUR MUM! and BEN 10’s REVENGE! are two stories, published a few days apart, which both deal with violent endings to Ben10 relationships. The graphic violence depicted in these stories is by no means unusual: it is typical of the “banality” of “ordinary” township crime and violence (Sandwith 2017) that is the tabloid’s stock in trade. What is interesting is the treatment of the violence and trauma, which is overtly narrativised: plunged directly into the story-like form, the reader is invited to imagine “the subjective experience of (the) victims” (Sandwith 2017: 187). The concentrated structure of the stories emphasises the women’s sudden reversal of fortune and the “radical disjuncture” (Sandwith 2017: 189) between their past pleasure and present misfortune.

“Ben 10’s revenge” is prefaced by a dramatic image of a burned house - the blackened ruin dwarfs the figure of the woman who stands before it. The report describes how her “raging Ben 10” “destroyed her house” and “stole her possessions.” The man’s “revenge” is a response to being told by his fifty-six-year-old lover “to pack his things” and “get out.” The report highlights the woman’s sudden change in circumstances: she is now “living in the backyard” of her now derelict “10-roomed house”; pitifully, she “(doesn’t) know what to do” and she “(needs) help” for she is “not working anymore.”

The second story opens with a bald and shocking statement: “Cops are looking for a Ben 10 who allegedly killed his lover with an axe yesterday morning.” In the accompanying image a young woman holds out a photograph of her mother who, like the daughter, looks directly at the reader. The composition of the women’s direct and unsmiling gaze demands the reader’s attention and response. Starkly, we are told that Nobengazi Matshoba, whose age is given as “50,” was “found dead in front of her new RDP.” The couple met when he lived in her “backyard shack” - the implication is that she was his landlady. But the relationship soured: the couple “had been fighting for the whole week” because she had “dumped him” and “told him to leave her property.” The description of the murder scene reveals the man’s violence: she is found “lying in a pool of blood,” there are “deep wounds on the back of her body” and an “axe was lodged in her head.” These grisly details contrast the description of the “good mum,” who “earned a monthly income” as a “domestic worker.” Her “six kids” and “three grandkids” are not only “unemployed” but now “left behind.” The “Ben 10,” also termed a “boyfriend,” is described as an “evil man” who “smoked drugs,” and “ran away with her bag, containing her money and cellphone.”

#### Facebook Comments Discussion

Both articles provoke strong reactions from readers, and the women involved are simultaneously pitied and blamed for the crimes that are committed. Compassionate comments construct older women as vulnerable to Ben 10s and warn them to “be careful” and “please stop dating Ben 10s,” as they are “very cruel” “spoiled brats” who “don’t want to work,” and “very jealous” men who are “not mature enough to handle a break up.” They are right to be concerned: intimate femicide is the leading cause of female murder South Africa (Mathews et al., 2014). In 2019 alone, South African Police Service (SAPS) crime statistics show that 38,656 cases of domestic abuse were reported, including GBH and murder (SAPS Crime Stats: 2019). These egregious statistics are linked to inequitable and patriarchal gender norms that validate the use of violence and the male control of women (Ratele 2008; DWYPD 2020).

Thus, the virulence of the *blame* directed at the women is noteworthy. Older women are vilified for being “desperate” and breaking social norms by taking younger lovers: “that is a taboo old lady” remarks one. The crimes against them are read as a satisfying warning: “Dats gud lesson to b learned by omagogo (old women) who dates young boyz” is a frequent sentiment. Such women are “disgusting” and can even “rape their grandsons.” They are “old meat,” and sex with them is “a sin” which “should b a crime.”

The real crime appears to be the older woman’s sexuality, which is constructed as predatory, as suggested in phrases such as “U wanted fresh young dick,” “after finishing him in bed u brought this to yourself” and “You was greedy for strong 4 5 (penis) why you complaining now.” For others, it is the combination of predatory sex and rejection that is offensive: “no one dumps a BEN10 and gets away with it after milking him out unless he say so.” This sentiment is frequently repeated in combination with reference to the material support enjoyed by the man: “(What) did u expect giving him candy then while he is enjoying ur money n ur old cake (vagina) u want to take that away?” “You gave him a home… then you chase him out after finishing him in bed” and “So what do you expect after they dump them, when bread is taken out of their mouth?” reiterate this theme.

Access to income and shelter in addition to sex are thus acknowledged as important components of such relationships in which the man is understood as in want, and the removal of this security is accepted by many as a motivation for their violence against their older and financially independent lovers. This explanation is complemented by the suggestion that the violence is provoked by the inversion of age-appropriate roles: “Monna ke Hlogo ya lapa (the husband is the head of the household)” argues one man, and “we were born to lead.” The problem arises when the older woman “shows her maturity over that man”; it is at this point that “he is going to behave bad.” Reflecting on the violence of men who “are powerless in relation to other males but at the same time members of a powerful gender group in relation to females,” Ratele (2008) foregrounds age (seniority) and occupation (the ability to earn an income) as central components of ideal “hegemonic” masculinity in Africa. Age is an important principle for the organization of gender relations in Africa, as it allows men to assume masculine authority (through initiation, work, marriage, becoming an elder, etc) (Ratele 2008: 534). However, it is employment that enables the realisation of these ideals, such as marriage. Linking violence to frustrated ideals of masculinity, Ratele (2008: 529) explicitly connects levels of male violence to “levels of unemployment, specifically unemployment in contexts of great income inequality,” such as those in South Africa.

But readers struggle to make sense of the verbal and physical violence. Punitive opinions are noted with shock: “some comments!! Some people a cruel” exclaims one reader, “Hv some sympathy 4 diz woman.” Another picks up the implicit logic of the comments and asks plaintively: “if I date a younger man than me, does he have a right to abuse or kill me?” This is no rhetorical question: “Please Mzansi, I’m waiting for your honest answers,” she pleads. “Domestic abuse is real. Irrespective of age,” agrees another, but cannot suggest a solution.

Interestingly, a remedy is given by a man who encourages older women to look for chivalrous younger lovers who won’t abuse them. He begins with the premise that older people still desire sex but don’t want sex with age-mates: “Gogos hv no choice” he argues, but to have younger lovers as they are no longer attracted to men their own age—and anyway older men are looking for “young girls.” His advice then is that women “shud choose a well-mannered ben 10 to avoid da situation like this one.” “Take yo time to search for those ben 10s,” he reassures them, “There are gud ones out there.”

### HIV/Aids

The incidence of HIV decreased significantly between 2012 and 2017 due to the rollout of ARV treatment. However, sexually active women of all ages remain more vulnerable than men to infection; while men are less likely to know their status and be on treatment, and thus more likely to risk infecting their sexual partners (HSRC 2017). In addition, older women are far less likely than younger women to insist on condom use (HSRC 2017). There is also strong evidence that inequality within sexual relationships, which is also linked to intimate partner violence, enhances the risk of HIV infection (Jewkes and Morrell, 2010: 1). Given this scenario, it is perhaps surprising that safe sex is not mentioned in stories about sexual relationships—rather, it becomes conspicuous in its relative absence. It never appears in the articles themselves, but emerges obliquely in occasional Facebook comments in which readers comment on the appearance and behaviour of the characters.

Thus, in response to Puseletso’s story, a reader points out the couple’s apparent ill health as interpreted from the photograph: “Bt i m worried about their health as dey all look weak.” Another agrees with this interpretation, and on this account describes the couple as “mad”: “this is pure madness, Puseletso wa teng a mme wa ipona gore o fedile Jang?” (Does she not see how thin and sick she looks?).” Amused, someone suggests that the couple look after their health better: “lol Mama looks very unhealthy. sum fruit + veggies Bco and A-Z might to the trick b4 being a sex slave.” Many others disgustedly comment that the couple look “dirty.” “Weak,” “thin” and “dirty” bodies are taken as a sign of illness. In this way the couple’s health is used as a means of censoring their sexual conduct. But a contrary opinion argues “If it works… Why NOT? This is a solution to the problems we face nowadays (pple killing for love, sti’s,etc.). More woman should follow in her footsteps.”

A similar conclusion is reached by a male reader of “MY BEN 10 IS A SUPERMAN”, who suggests that the relationship is beneficial as it keeps the man safe from sexually transmitted infections: “i think it’s much safer for this guy” he states. This is because “magogo is not sexually active any more he is her only lover which means less chance of getting std’s”. In other words, a younger woman with more than one lover would increase the risk of infection. In either case, the man is understood as being at risk from the woman.

Concern about safe sex is seen in comments on “I’M A BEN 10 TO MAKE MY LIFE BETTER, a letter to “Mizz B,” the paper’s advice column. A male reader begins a debate by joking that “using a condom is like standing outside a club but telling people you went in.” In other words, condom users are lying to others—and to themselves—if they say sex with a condom is the same as sex without one. Metaphorically, the man remains outside the “club,” and must imagine, rather than experience, what goes on inside. Several responses agree and laugh at the analogy: “Loooolz (laughter) responses one, while another comments several hours later that “I’m still laughing.” However, one commentator explicitly calls out the laughter: “don’t fool people,” he scolds, “as long it gets u to de same destination as de flesh guy no diff.” He emphasizes that safe sex using a condom still ensures sexual satisfaction, while agreeing that using one “may giv prblm.” He then uses a metaphor to remind readers of the reality of HIV: “but wit dis of today (but with this situation today) shayis gubhu kuphumumsindo klaar (beat the drum to the sound, period).” In other words, let your actions suit the situation.

## Conclusion

Older, financially independent, working class South African women who risk asserting their autonomy and sexuality by taking younger lovers in a patriarchal context where rights are not guaranteed face a range of significant obstacles. These differ substantially from the constraints encountered by older women in northern spaces. Global postfeminist discourse is generally understood as advocating that older women age “appropriately,” re-inscribing them within patriarchal gender relations through physical makeovers, normative heterosexuality and consumption within neoliberalism. It demands that older women work hard to attain and conform to an ideal feminine appearance and sexuality, while taking a context of financial independence and “rights” for granted.

The *Daily Sun* Ben 10 narratives and Facebook comments point to a far more complex local gender terrain. By publishing Ben 10 stories, *Daily Sun* marks these relationships as intrinsically noteworthy, and the large number of Facebook comments they generate substantiate this interpretation. We suggest that this is because they call into question the fundamental premises of gender relations in our postcolonial space. Nor can women’s rights be taken for granted: the stories suggest that older, independent and sexually active women in working class social spaces who dare to take younger lovers in a context of profound economic inequality risk social censure, violence and the possibility of HIV infection, significant constraints on their autonomy.

*Daily Sun* marks successful Ben10 relationships as sensational and slyly ridicules both the woman and the men involved, such as by emphasising their age difference and depicting them dressed inappropriately (for example, a frilly hat). However, they simultaneously represent successful relationships as positive for the women and refrain from condemning their choices, which are constructed as in keeping with modern mores. To this end, the presentation of the stories is designed to provoke discussion, in keeping with the paper’s community-building objectives (Boshoff 2017). Contrastingly, reader’s comments on such relationships range between wholehearted support and utter condemnation. In the first case, the older woman is held up as a role model for other women who would like to be sexually active and independent. Readers draw on a discourse of rights to justify her choice and to problematize customary gender relations which subordinate women. In the second, she is seen as a horrifying aberration, deemed mad and immoral, and shaming the husband. For such readers, the “rights and wrongs” of the woman’s choice is more important than her “rights.” More moderate and objective readers attempt to rationalise older women’s choices as individual responses to changing life and physical circumstances, without reflecting on the systemic issues at play.

Reports of unsuccessful relationships that end violently are compassionate of the women even as they draw attention to the horror of their plight - the paper gives them or their families ample space to explain their predicament and garner sympathy and support from readers. Some readers do sympathise, linking the crime to the wider problem of gender-based violence. They condemn the man’s actions while warning other women to take care of themselves and steer clear of such. Others, however, construe the man’s violence as an understandable—even expected—response to his social subordination and the woman’s predatory sexuality. The woman’s death or loss of property is seen as her just deserts. At the same time, readers condemn the men, for their lack of employment and their dependence on women is read as a despicable failure of masculinity. Neither successful nor unsuccessful relationships address HIV and safe sex. The general guardedness of the comments on the issue suggest that the topic is taboo in public fora such as the *Daily Sun* Facebook page. Most comments on the topic appear aimed at provoking laughter and ridicule. A few readers dare to suggest seriously that men protect themselves from infection by women. No comments are made to suggest that women protect themselves from infection by men.

Our analysis suggests that as researchers interested in understanding the purchase of postfeminist discourse in southern contexts we must pay close attention to three linked concerns. The first is history: postfeminist identities are advocated and adopted in contexts shaped by particular experiences of colonialism and liberation. This history includes an ongoing struggle to order patriarchal gender relations. The second regards how men and women respond to postfeminism given their disposition within this historical formation. As situated subjects they work with a range of competing and complementary discourses of which postfeminism is but one. Third, where do we look for evidence of postfeminism’s reach? Local popular culture forms may offer new insights into how global discourses are translated for and received by local audiences. Postfeminist theory could be enriched by taking into account the ways in which postfeminist ideals of women’s economic autonomy, sexuality and choice of partner emerge from and play out within particular historical and social contexts. South Africa’s colonial and post-apartheid history of gender-relations, including rights, violence and HIV, and the suppression of sexually and financially independent women provides the wider context in which the discourse of postfeminism is encountered and understood within the *Daily Sun*. By paying attention to this history, which weighs so heavily on older, working class women, postfeminist theory can more carefully map both the “edges” of the discourse (Gill 2017: 609) and the means by which it entrenches itself and evolves within particular locales.

## Daily Sun Articles

BEN 10’s REVENGE! Daily Sun April 18, 2017
http://bit.ly/2pNJC6Z
EVIL BEN 10 KILLED OUR MUM! Daily Sun April 20, 2017
http://bit.ly/2oXtanq
‘I’M A BEN 10 TO MAKE MY LIFE BETTER’ Daily Sun September 10, 2016
http://bit.ly/2bz7sM8
‘I LIVE WITH MY BEN 10 AND MY HUBBY!’ Woman takes a second husband for sex! Daily Sun March 12, 2015
http://bit.ly/1HIP9PB
MY BEN 10 IS A SUPERMAN Daily Sun March 24, 2017
http://bit.ly/2nL3oTb

## Data Availability

The original contributions presented in the study are included in the article/Supplementary Material, further inquiries can be directed to the corresponding author.
